# Black Bear Behavior and Movements Are Not Definitive Measures of Anthropogenic Food Use

**DOI:** 10.3390/ani13050950

**Published:** 2023-03-06

**Authors:** Don W. Hardeman, Hannah B. Vander Zanden, J. Walter McCown, Brian K. Scheick, Robert A. McCleery

**Affiliations:** 1 Department of Wildlife Ecology and Conservation, University of Florida, 110 Newins-Ziegler Hall, Gainesville, FL 32611-0430, USA; 2 Fish and Wildlife Research Institute, Florida Fish and Wildlife Conservation Commission, 1105 Southwest Williston Road, Gainesville, FL 32601-9044, USA; 3 Department of Biology, University of Florida, P.O. Box 118525, Gainesville, FL 32611-8525, USA

**Keywords:** stable isotopes, *Ursus americanus floridanus*, food conditioning, wildlife conflict

## Abstract

**Simple Summary:**

Growing human populations and development into previously undisturbed areas have increased human wildlife conflict with carnivores and particularly bears. Wildlife managers often remove bears from areas of human development under the assumption that they will become accustomed to eating human food and are more likely to cause conflicts. Using measures of nitrogen and carbon derived from bears hair, we determined if bears showed patterns of human food consumption. We found that observations of bears moving around developed areas were not always strong predictors of prolonged patterns of human food consumption. Our findings suggest managers and the public should not assume that bears found in and around developed areas are accustomed to human foods and likely to cause continued conflicts.

**Abstract:**

Increasing human–bear conflicts are a growing concern, and managers often assume bears in developed areas are food-conditioned. We examined the relationship between human–bear conflicts and food conditioning by analyzing isotopic values of hair from black bears (*Ursus americanus floridanus*) involved in research (*n* = 34) and conflicts (*n* = 45). We separated *research* bears into *wild* and *developed* subgroups based on the impervious surface within their home ranges and separated conflict bears based on observations of human food consumption (*anthropogenic* = observations; *management* = no observations). We initially assumed wild bears were not food conditioned and anthropogenic bears were. However, using isotopic values, we classified 79% of *anthropogenic* bears and 8% of *wild* bears as food conditioned. Next, we assigned these bears to the appropriate food conditioned category and used the categorizations as a training set to classify developed and management bears. We estimated that 53% of *management* bears and 20% of *developed* bears were food conditioned. Only 60% of bears captured within or using developed areas showed evidence of food conditioning. We also found that δ13C values were a better predictor of anthropogenic foods in a bear’s diet than δ15N values. Our results indicate that bears in developed areas are not necessarily food conditioned and caution against management actions based on limited observations of bear behavior.

## 1. Introduction

Growth in human populations and development over the last half century, as well as increases in some generalist wildlife populations, have increased interactions between humans and wildlife [1,2,3,4]. Certain wildlife species have been able to adapt to newly human-dominated landscapes where they use anthropogenic food sources (e.g., garbage, pet food, crops, livestock, wildlife feeders [5]) and share space with humans [6,7,8]. For example, bears (*Ursus* spp.) often traverse human-dominated landscapes and can exploit human resources [9,10,11,12], but they typically avoid areas with more human development [13] that they likely perceive as risky [14]. However, competition-based dispersal, availability of seasonal foods, and physiological periods with high-energy requirements (such as hyperphagia [15]) may contribute to the use of developed areas despite the risks [16,17].

In recent decades, wildlife managers have reported increased human–bear interactions across the human-dominated landscapes of North America [18,19,20,21]. The dominant driver behind these interactions appears to be the availability and consumption of anthropogenic foods by bears [5,17,19,22]. Bears that use anthropogenic foods often alter their movement and foraging behaviors to find these calorie-rich foods [23,24,25].

The American black bear (*Ursus americanus*) is the most common bear species in North America, and the majority of human–bear interactions involve this species. Black bears are intelligent, behaviorally plastic, have broad tolerances for food and vegetation types, and can move over large areas [22,26]. These traits can make them susceptible to habituation and food conditioning [27,28], learned behaviors that form when the association between developed areas and risk is reduced and an animal equates human development with food [21,29,30]. Naive bears presumably enter developed areas for the first time during exploratory movements [17,30,31], then, over repeated visits, they become habituated and/or food-conditioned [5,29]. The degree of a bear’s food conditioning is influenced by the availability of natural and anthropogenic food sources [32], tolerance of humans [29], and reproductive status [33], with highly food-conditioned bears more likely to have negative human interactions (e.g., cause property damage and injuries to humans [32,34]).

Traditionally, any incident of a bear occurring in or near human developments would be classified as a conflict due to the potential for injuries to people or damage to property [35], with the view that repeat offenders should be translocated or euthanized [11,36]. A common underlying assumption behind the classification of a conflict animal is that a bear in a developed area is either food-conditioned or is likely to become food-conditioned. In contrast, Elfstrom et al. [35] found that limited observations of any particular bear were insufficient to discriminate between food-conditioned and non-food-conditioned brown bears (*U. arctos*); however, a bear’s movements associated with human development might be more predictive [37]. Additionally, other factors, such as the time of year, abundance of natural foods, and the density of bears, can influence a bear’s movements around human development [29]. For example, a bear may periodically access human-dominated areas that it would normally avoid during dispersal [35], hyperphagia [16], years of low natural food availability [12,17], or when natural foods occur near development is abundant [17,24]. Accordingly, management agencies recognize a need for a more adaptive, contextual, and data-driven approach in their response to bears in and around human development [21,35,38].

Stable isotope analysis is a powerful but coarse measurement that can be used to understand animal diet and potentially inform management policies [39,40]. Carbon and nitrogen stable isotopes in animal tissue, reported as δ^13^C and δ^15^N values, respectively, are assimilated via dietary foods and reflect the resources consumed [40,41,42,43]. Hair can be collected invasively and non-invasively [44,45], and stable isotope analysis of hair can be used to determine if bears had consistently relied on human-sourced foods, which we interpret as an indication of food conditioning. Bears with elevated δ^13^C values in areas without naturally occurring C_4_ plants likely have a diet subsidized by corn or sugar cane via anthropogenic food sources [46,47]. Higher δ^15^N values are indicative of trophic levels and could identify the consumption of animal-protein-based foods [47]; such values are well suited for identifying anthropogenic foods in areas where black bears have a predominantly plant-based diet [48].

As black bears continue to increase in abundance and expand their range, it is essential that wildlife managers find additional tools to interpret bear behavior and assess risk of human-bear conflicts. Accordingly, our objective for this study was to examine the relationship between human–bear conflicts and food conditioning in bears by direct but limited observations during human–bear conflicts and seasonal use of developed areas to develop a predictive model of food conditioning. We predicted that direct but periodic observations of bear behavior would be of limited value when predicting isotopic values indicative of food conditioning. Alternatively, longer-term information on a bear’s presence around human development is likely to be more predictive [37].

## 2. Materials and Methods

### 2.1. Study Area

**Figure 1:**
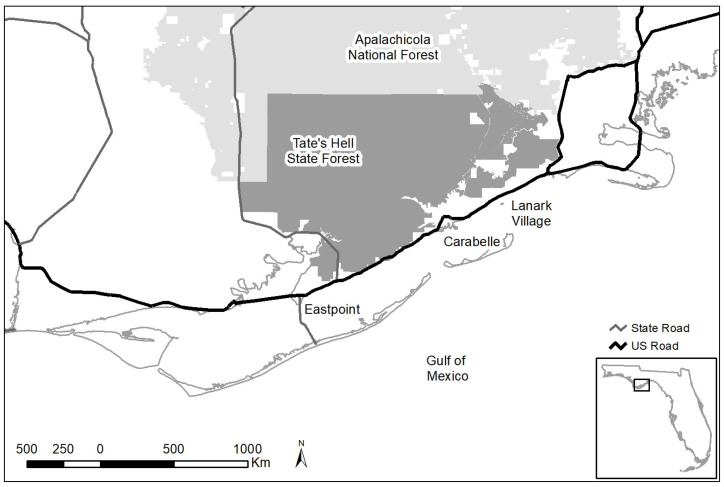
The study area consisted of Tate’s Hell State Forest (darker shaded area) and neighboring commercial timberlands. The towns mentioned in text are shown as are major highways and Apalachicola National Forest boundaries (lighter shaded area).

We conducted our study on the Tate’s Hell State Forest and neighboring private lands in the eastern panhandle of Florida (Figure 1), a region that supports the second largest subpopulation (1060 individuals) of black bears in Florida [49]. Tate’s Hell State Forest is the second largest contiguous piece of state land in the panhandle of Florida and is composed of 819 km^2^ of contiguous wet flatwoods, wet prairie, sandhill, upland hardwood, floodplain swamp, scrub, and titi thickets. The forest is heavily ditched, a remnant of past commercial timber production, and it is characterized by poorly drained soils. The forest sustains a high level of recreational activity and is open to off-road vehicles all year. The towns of Carrabelle (pop. 2778), Eastpoint (pop. 2337), and the smaller, unincorporated Lanark Village are on US road 98 along the Gulf of Mexico, which formed the southern border of the study area; the Apalachicola National Forest is adjacent along the entire northern border. Except for rural residents, much of the private lands are managed for commercial timber and leased for hunting (currently, bear cannot be legally hunted in Florida).

The local climate consists of hot, humid summers, and short, cool winters with average daily temperatures ranging from 8 °C to 18 °C in January and 24 °C to 33 °C in July. Precipitation in the area is highly variable and primarily falls in the summer and early fall [50].

### 2.2. Overview

**Figure 2:**
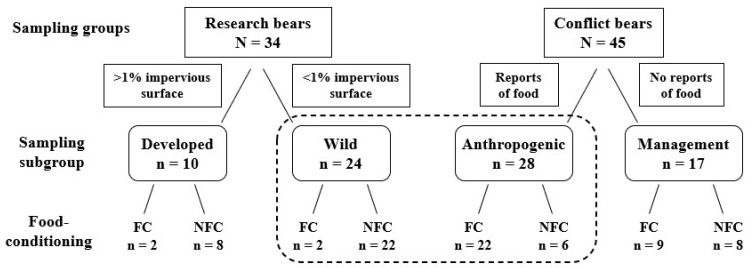
The conceptual framework for assessing the relationship between food conditioning and bears grouped by behavior or movements and the results of stable isotope and linear discriminant analysis (LDA) analyses. The development of groups and subgroups were based on movements in black bears as determined by the percent impervious surface within individual 95% MCP fall home ranges or bear behavior reported when human-bear conflicts were reported. The dotted box highlights the training data for the LDA analysis.

We collected hair from bears from 2015 to 2017 to determine if food conditioning, assessed by stable isotopes in hair, could be predicted by proximity to developed areas or a direct observation of bears eating anthropogenic foods. We categorized bears into different groups and subgroups based on the data available for each individual (Figure 2). We classified the radio-tagged *research* bears, captured only during summer months, as *wild* or *developed* based on their home range. We classified *conflict* bears removed from the population by managers (killed or translocated during any month of the year) as *anthropogenic* if observed consuming anthropogenic foods or *management* if removed without being observed consuming anthropogenic foods.

### 2.3. Classification of Bears

#### 2.3.1. Capture and Monitoring of *Research* Bears

*Research* bears used in this study were part of a larger project conducted by the Florida Fish and Wildlife Conservation Commission (FWC) to study the demographics of the Apalachicola bear subpopulation, for which only female bears were radiocollared. For that study, we captured bears between May and July from 2016 and 2017 using cage traps, culvert traps, and spring-activated Aldrich foot snares (Margo Supplies Ltd., High River, Alberta, Canada), following Johnson and Pelton [51], as modified by Scheick et al. [52]. All traps were set on public lands or nearby commercial timberlands. We immobilized bears with a 1:1 mixture of tiletamine hydrochloride and zolazepam hydrochloride (Telazol) administered at 4 mg/kg of estimated body weight (dosage ranged 2.0–10.5 mg/kg) via a CO_2_ powered dart gun (Telinject, Inc., Arleta, CA, USA). We processed each individual and collected hair samples from the dorsum. We fitted 37 subadult and adult female bears with iridium GPS satellite-tracking collars (Lotek Wireless Inc., New Market, ON, Canada) programmed to collect fixes at 2-h intervals for two years post-capture.

#### 2.3.2. *Research* Bears

To categorize our *research* bears as *wild* or *developed,* we used impervious surfaces (e.g., roofs, paved roads, parking lots, compacted soils) that prevent water from entering soils, as a common measure of human development [53,54]. Specifically, we calculated the proportion of impervious surface across the home range area of each individual during the months associated with hyperphagia in Florida (1 September–30 November) in 2016 and 2017. This physiological state represents the period of greatest bear activity [15] and therefore increased the likelihood that these bears would enter human development for food resources. Our dataset of *research* bears included 34 individuals after we removed three individuals that did not have locations within our study area during the majority of the three-month period. We also removed all GPS fixes that were not three-dimensionally validated (four or more satellites) to maintain data quality. We estimated fall home range areas by 95% minimum convex polygon (MCP) using the *adehabitatHR* package in program R [55].

To calculate the amount of impervious surface, we extracted the values from an impervious surface raster file [56] and quantified the mean impervious surface within the range of each bear using the *raster* package [57] on the R platform [58]. The mean impervious surface across all home ranges was 0.99%, with a standard deviation of 1.1 (Figure S1). We selected an impervious surface level just above the mean (1%) as the cutoff and classified bears with ≤1% impervious surface in their range as *wild* and those >1% as *developed* (Table S1). We chose this cutoff based on observations that bears with >1% of their MCP containing impervious surface altered their behaviors to human approach (unpublished data). To ensure that MCP was an appropriate measure of bear’s exposure to human development, we compared the amount of impervious surface within each bear’s 95% MCP to the proportion of its GPS locations with an impervious surface > 1% divided by locations with an impervious surface < 1% (to estimate the amount of time spent there). Finding both measurements highly correlated (0.76; *p*-value ≤ 0.01), we proceeded using MCP as a measure of exposure to development.

#### 2.3.3. *Conflict* Bears

We used hair samples and data collected from 45 bears captured by FWC staff, from across Florida, in response to human–bear conflicts between July 2015 and November 2017. These bears were captured using cage or culvert traps similar to those used for the research bears; however, they were set in specific areas where conflict behavior had been reported. We used written reports that summarized the direct observations by the complainant and the responding FWC biologists to classify these *conflict* bears as *anthropogenic* or *management*. We classified 28 bears as *anthropogenic* because reports for each of these bears indicated they were observed eating anthropogenic food, usually garbage, at least once. The stated capture reason for most of these bears was getting into garbage, but eight were also considered a human safety risk based on the bear’s behavior (not for repeatedly consuming garbage). Two family groups were included (Bear ID 17355 was the mother of 11-month-old cubs 17356 and 17357; Bear ID 17523 was the mother of a dependent yearling 17522), and 46% were cubs or subadults (aged ≤ 2.9 years). We classified 17 bears as *management* bears because they were captured and removed for conflict behavior (one attacked a human, seven caused property damage, and nine were considered a human safety risk), but without direct observation that they ate anthropogenic food. Fifty-nine percent of the *management* bears were juveniles.

### 2.4. Stable Isotope Analysis

We used stable isotope analysis to determine the δ^13^C and δ^15^N values of each individual to establish its dietary status. We assumed that all *anthropogenic* bears were food conditioned (FC) because they were seen eating human food and that all wild bears were not food conditioned (NFC) because they were rarely present in the human-dominated landscape. There were no commercial agricultural fields in our study area, but automated corn feeders for deer were prevalent on private lands, and small amounts of corn could be available from residential gardens. We tested these assumptions by comparing the isotopic values of the two groups. We then used these results to reclassify bears more appropriately as FC or NFC and establish a training dataset capable of predicting the FC or NFC classification. Next, we used our training dataset to test the prediction that bears with movements that overlapped the human-dominated landscape (*developed research bears*) or observed in the human-dominated areas but with no direct observation of anthropogenic food consumption (*management conflict bears*) would display isotopic values consistent with FC classifications.

A bear’s hair grows in roughly linear fashion over the period when they are active [59,60]. During periods of growth, hair integrates assimilated protein, carbohydrates, and fats [45] in metabolically inert keratin, thereby preserving the isotopic information indefinitely [61]. The ability to determine average diet during the period of growth (i.e., whole growth analysis) makes hair useful in stable isotope analysis [44]. Bears undergo a single molt per year that occurs between early May and fall, depending on the quantity and quality of available forage [62]. Therefore, we assumed the full-length guard hairs collected during summer research prior to molting represented the isotopic composition of the diet from the previous year’s molt until the time of capture in the current year. Similarly, full-length hair collected from *conflict* bears in or prior to August was assumed to represent the previous year’s diet, while hair collected in or after September represented the capture year’s diet (August to time of capture).

We cleaned hair samples with a 2:1 chloroform-methanol solution to remove surface oils before air-drying them [47,63,64]. We cut hairs at the base and weighed samples in tin capsules and sealed them for isotopic analysis. Values of δ^13^C and δ^15^N were determined using continuous flow isotope ratio mass spectrometry using a Thermo-Scientific DeltaV (Waltham, MA) Advantage Isotope Ratio Mass Spectrometer connected by a ConFlo II interface to a Thermo-Scientific Carlo Erba NA 1500 CNHS elemental analyzer at the Stable Isotope Mass Spectrometry Lab at the University of Florida. Stable isotope values were expressed as per mil (‰) in standard delta (δ) notation:

(1) $$\delta X=(R_{\mathrm{sample}}/R_{\mathrm{standard}})-1$$

where δX is ^13^C or ^15^N, and R is the corresponding ratio of heavy-to-light isotopes (^13^C/^12^C or ^15^N/^14^N) in the sample. We reported results as ratios relative to international reference standards Vienna Peedee Belmnite (V-PDB) for carbon and to atmospheric N_2_ (AIR) for nitrogen. The precision for δ^13^C and δ^15^N values was ±0.06‰ and 0.11‰ respectively, based on 5 analyses of USGS40.

### 2.5. Statistical Analyses

We used MANOVA to test if bears classified as *anthropogenic* bears based on reported human-sourced food use and *wild* bears based on impervious surfaces had different isotopic values. We then performed linear discriminant analysis (LDA) to assess our ability to distinguish *anthropogenic* bears and *wild* bears (Table S2) based on the δ^13^C and δ^15^N values of their hair [65,66]. We used the posterior probability predicted by the LDA to reclassify the bears categorized as *wild* or *anthropogenic* into new groupings, FC or NFC, based on their respective isotopic values. We then used the reclassified FC bears and NFC bears to establish a training dataset capable of predicting the FC or NFC status of the *developed* and *management* bears. To evaluate the accuracy of the training data to predict group assignments we used leave-one-out cross-validation (LCV [67]). Next, we used the training data to assign *developed* and *management* bears to a category (FC or NFC) based on the highest posterior probability predicted by the LDA with LCV [65]. We used the structure coefficients from the LDA to determine whether carbon or nitrogen was better at discriminating between FC and NFC bears. We performed all statistical analyses on the R platform [58].

## 3. Results

We determined the isotopic composition of hair, based on the δ^13^C and δ^15^N values, from 79 individual bears. Samples collected from *anthropogenic* bears *(n* = 28, Figure 2, Table S2) had a δ^13^C mean = −20.33, SD = 2.02, δ^15^N mean = 6.00, and SD = 1.38, and *wild* bears (*n* = 24, Table S2B) had a δ^13^C mean = −23.57, SD = 1.46, δ^15^N mean = 4.36, and SD = 0.96. *Anthropogenic* and *wild* bears together had a δ^13^C mean = −21.90, SD = 2.35, and δ^15^N mean = 5.21, SD = 1.44; *management* bears (*n* = 17) had δ^13^C mean = −21.25, SD = 1.92; δ^15^N mean = 4.99, SD = 1.25, Table S2); and *developed* bears (*n* = 10) had a δ^13^C mean = −22.84, SD = 1.79; δ^15^N mean = 4.83, SD = 0.91, Table S2).

**Figure 3:**
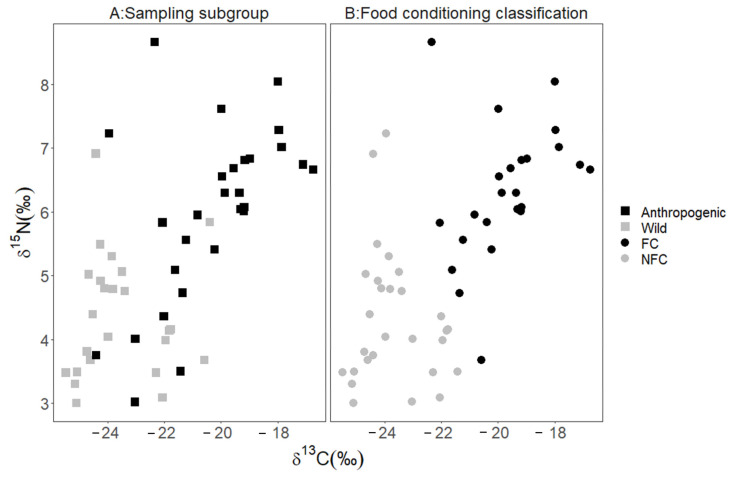
Stable isotope values (δ^13^C and δ^15^N) for bear hair samples. Isotope values for bears captured throughout Florida from 2015 to 2017 comprise the *anthropogenic* bears (■) sampling subgroup and bears captured in and around Tate’s Hell State Forest, Florida, USA from 2016–2017 comprise the *wild* bears (■) subgroup. Higher values indicate more human-sourced food. The original classification, plot (**A**), depicts classifications based on amount of impervious surface (wild bears) and observed conflict behavior (*anthropogenic* bears). The food conditioned classification plot (**B**) depicts the same individuals reclassified via a linear discriminant analysis with leave-one-out cross validation; six *anthropogenic* bears and two *wild* bears were reclassified to predict food conditioning FC (⬤) and not food conditioned NFC (⬤).

Using the results of the MANOVA, we established that *anthropogenic* bears and *wild* bears were significantly different groups (multivariate F (1,2) = 22.87, *p* < 0.001) prior to applying the discriminant analysis. We used LDA with LCV to determine that 79% (*n* = 22) of the *anthropogenic* bears (high δ^13^C and δ^15^N values) were classified as FC bears and the remaining 21% (*n* = 6; low δ^13^C and δ^15^N values) were reclassified as NFC bears (Figure 2 and Figure 3). A larger proportion of *wild* bears, 92% (*n* = 22), were classified as NFC bears, with the remaining 8% (*n* = 2) reclassified as *FC* bears (Figure 3). The classification rate for our model was 85%, which suggests high predictive capacity using δ^13^C and δ ^15^N values, supporting their use as training data.

**Figure 4:**
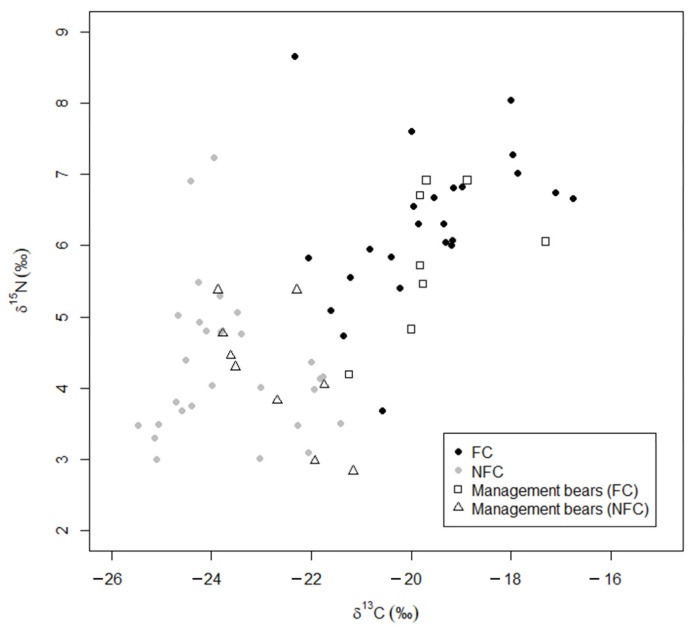
Stable isotope values (δ^13^C and δ^15^N) of *management* bears predicted by linear discriminant analysis as food conditioned (FC; ◻) or not food conditioned (NFC; △) compared to the training data (reclassification from (Figure 3B) showing the training data that reclassified *anthropogenic* and *wild* bears as food conditioned FC (⬤) and not food conditioned NFC (⬤). Higher values indicate more human-sourced food.

**Figure 5:**
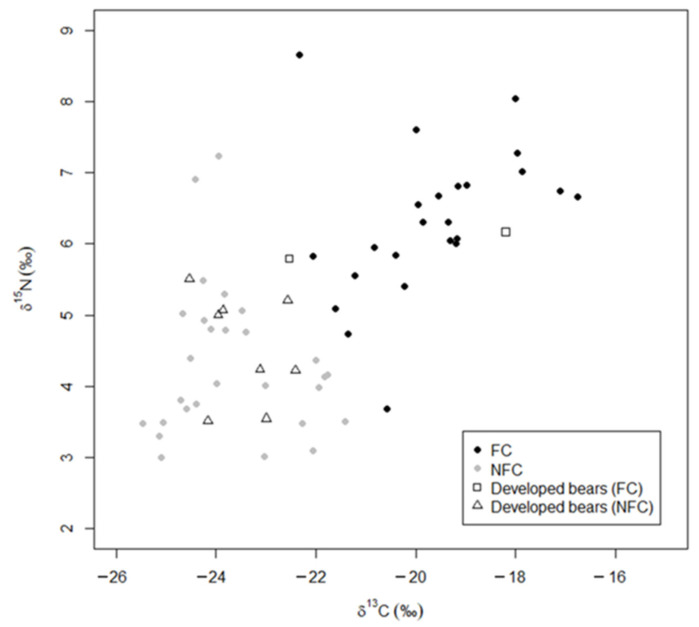
Stable isotope values (δ^13^C and δ^15^N) of *developed* bears predicted by linear discriminant analysis as food conditioned (FC; ◻) or not food conditioned (NFC; △) compared to the training data (reclassification from (Figure 3B) showing the training data that reclassified anthropogenic and wild bears as food conditioned FC (⬤) and not food conditioned NFC (⬤). Higher values indicate more human-sourced food.

The data on *wild* and *anthropogenic* bears, including those reclassified as FC or NFC, were used as training data to determine the FC and NFC status for the remaining two subgroups. We predicted that 53% of *management* bears were FC bears, and the remaining 47% were NFC bears (FC: *n* = 9; NFC: *n* = 9; Figure 4), and we predicted that 20% of the *developed* bears were FC, and the remaining 80% were NFC (FC: *n* = 2; NFC: *n =* 8; Figure 5). We used the structure coefficients from our LDA to determine that δ^13^C values predicted anthropogenic foods in a bear’s diet 96% better than δ^15^N values, although FC bears were more likely to have higher values in both δ^13^C and δ^15^N than NFC bears (Figure 3).

## 4. Discussion

Our results indicate that bears in developed areas are not necessarily food conditioned and, in fact, not all bears observed consuming anthropogenic foods had isotopic values indicative of food conditioning. Of the 55 bears that had been captured in or near developed areas, including those from the *conflict* group and the *developed* subgroup, 40% (22 of 55) lacked evidence of food conditioning. Even among the 28 *anthropogenic* bears that had been directly observed eating human-sourced foods, 21% (six) were NFC. For predictive power, <1% impervious surface as determined by telemetry data was a better predictor of food conditioning status than direct but limited observations (92% of *wild* bears were NFC vs. 79% of *anthropogenic* bears were FC). However, increased impervious surface area was not associated with increased food conditioning, as seen by NFC bears with 5% impervious surface (Table S1).

We also determined that δ^13^C values were a better predictor of food-conditioning status than δ^15^N values for bears in Florida, although FC bears usually had both higher δ^13^C and δ^15^N values than NFC bears. A few NFC *wild* bears had high δ^15^N values relative to others in the subgroup, and most δ^15^N values from all subgroups were higher than that of terrestrial browsers, perhaps indicating some use of carrion [43,68]. Such naturally occurring animal protein would reduce the ability of δ^15^N values to identify FC status.

The isotope values indicative of food conditioning would require repeated and consistent consumption of anthropogenic food sources over time during the diet year [47]. Dispersal behavior and exploratory food searching would explain why bears accessing developed areas for brief periods might not consume anthropogenic foods or consume them consistently enough to display isotopic values indicative of food conditioning [29,30]. Due to limited sample sizes, we were not able to further subset our data to compare *conflict* bears of different sexes and ages. However, more than half of the *conflict* bears analyzed in this study were subadults, supporting the hypothesis that exploratory movements bring naive bears into developments [35,69]. Additionally, some NFC bears in our study that were in or near developed areas may have been new to developed areas, or they may have switched their foraging behavior between anthropogenic and natural foods. Johnson et al. [17] found a negative correlation between availability of natural foods and reliance on anthropogenic foods and corresponding shifts in bears’ use of developed areas.

Isotope values indicated that only 53% of *management* bears were food-conditioned (Figure 2 and Figure 4), but *conflict* bears in this study were removed for specific behaviors, not because of an assumption of food conditioning. For many years, the public in our study area has reported bears eating acorns in residential yards (FWC unpublished data), and our results suggest that, even when in developed areas, some bears rely on natural rather than anthropogenic foods. Whereas it was traditionally assumed the nuisance bears were food conditioned [47] managers have pivoted to assessing the risk to human life and property based on season, availability of natural foods, and the bear’s age, sex, and behavior (level of aggression, repeated conflicts, etc. [21]). However, information on a bear’s history is rarely available, and it is hard to determine if recurring conflicts in the same areas were caused by the same bear.

Seasonal or annual shifts in foraging areas may explain the 8% (two out of twenty-four) of *wild* bears in our model that were reclassified as FC. Although we do not know the movements of the two FC bears (F605 and F608; Tables S1 and S2B) during the year prior to their capture, the time period for which the isotopic values reflected their diet, we examined the movement data from 2016 to 2017 (unpublished FWC data). We determined that, in the fall, they had moved with their cubs into areas with scattered homes (but not developed according to our 1% impervious surface category) where they could have accessed anthropogenic food sources, stayed in that general area for the winter, and then returned to their normal summer home range area in spring. In fact, F605 had been photographed with her cubs at a feeder (FWC unpublished data). Such movements suggest that, while living farther from developments might reduce the odds that a bear is food conditioned, few bears in Florida live in areas where people or human-sourced foods are absent. These seasonal shifts may also explain why bears in family groups that were captured together during a human–bear conflict did not all show the same FC status. Bear ID 17355, captured in January, was not classified as food conditioned, but both of her nearly 11-month-old cubs were, so it seems likely that she brought her cubs to anthropogenic food sources that she had seldom or only recently used prior to her capture. In contrast, Bear ID 17253 and her dependent yearling were both food conditioned.

Implementing proactive management strategies (i.e., garbage management and educational programs) are effective means of reducing human–bear conflicts [19,21,70]. Johnson et al. [70] found that 60% compliance with keeping trash secured reduced human–bear conflicts by 60%. Removing readily available trash should also reduce food conditioning [47], although that is more difficult to prove empirically. There appears to be a lag between reduced conflicts and increased tolerance of people for bears, even when that reduction was caused by active participation in making trash less accessible to bears [71].

## 5. Conclusions

Expanding human development into more natural areas is expected to continue, increasing the likelihood of human–bear interactions, and exacerbating both real and perceived risks [31]. The complexity of the issues surrounding human–bear interactions requires wildlife researchers and managers to develop a more comprehensive understanding of the factors shaping bears’ diet, movement, and behaviors [31]. Kirby et al. [64] reported that each 1‰ increase in δ^13^C increased the likelihood that a bear would cause human–bear conflicts by 60%. We recognize that managers cannot wait for stable isotope analysis to determine the most appropriate management actions. Still, our findings illustrate the benefit of stable isotope analysis before classifying bears as food conditioned and they validate the importance of not assuming all bears observed in neighborhoods, or even directly observed feeding on human-provided foods, are food conditioned. Our results show that a bear’s presence in a developed area does not necessarily indicate it is food-conditioned. This should be taken into consideration when making management decisions and incorporated into educational materials. A public that has a better understanding of how anthropogenic foods affect the gradient of a bear’s learned behavior would be a more helpful partner with management agencies in reducing human-bear conflicts.

## Data Availability

The data used in the paper are provided in Supplemental Tables S1 and S2.

## Supplementary Materials

The following supporting information can be downloaded at: https://www.mdpi.com/article/10.3390/ani13050950/s1, Figure S1: Average percent of impervious surface available within the fall home range areas (1 September–30 November) for each of the 34 female bears in the *research* group, Table S1: Summary of each *research* bear’s minimum convex polygon (MCP), mean impervious surface (% Imp. surf.) across the fall home range area, and the subgroup where each female was placed based on impervious surface. This subgroup determined the original assumption of food conditioning. The MCP values are reported in kilometers squared (km^2^) rounded to the nearest hundredth. Table S2: Individual, mean and SDs of stable isotope (δ^13^C and δ^15^N) values of *anthropogenic* (A) and *management* (C) bears sampled throughout Florida during 2015–2017 and *wild* (B) and *developed* (D) bears sampled in and around Tate’s Hell State Forest, Florida, USA, 2016–2017. Subadult age class includes bears aged 1–2.9 years. Diet-year denotes the capture year or prior year for *anthropogenic* and *management* bears, depending on month of capture, and the year prior to capture for *wild* and *developed* bears. LDA classification denotes the two categories (not food conditioned [NFC] or food conditioned [FC]) based on the linear discriminant analysis with leave-one-out cross validation. P-FC is the probability of each individual being food conditioned. While all *anthropogenic* bears were initially assumed to be FC, and all *wild* bears were initially assumed to be NFC, some bears from each subgroup, in bold, were reclassified based on the LDA outputs.
