# Supplementary material for: Black Bear Behavior and Movements Are Not Definitive Measures of Anthropogenic Food Use
Source: Animals (Basel). 2023 Mar 6;13(5):950. doi: 10.3390/ani13050950 (PMC10000168; doi:10.3390/ani13050950)
Supplement: Supplementary file 1 [file animals-13-00950-s001.zip › animals-2227923-supplementary.pdf]

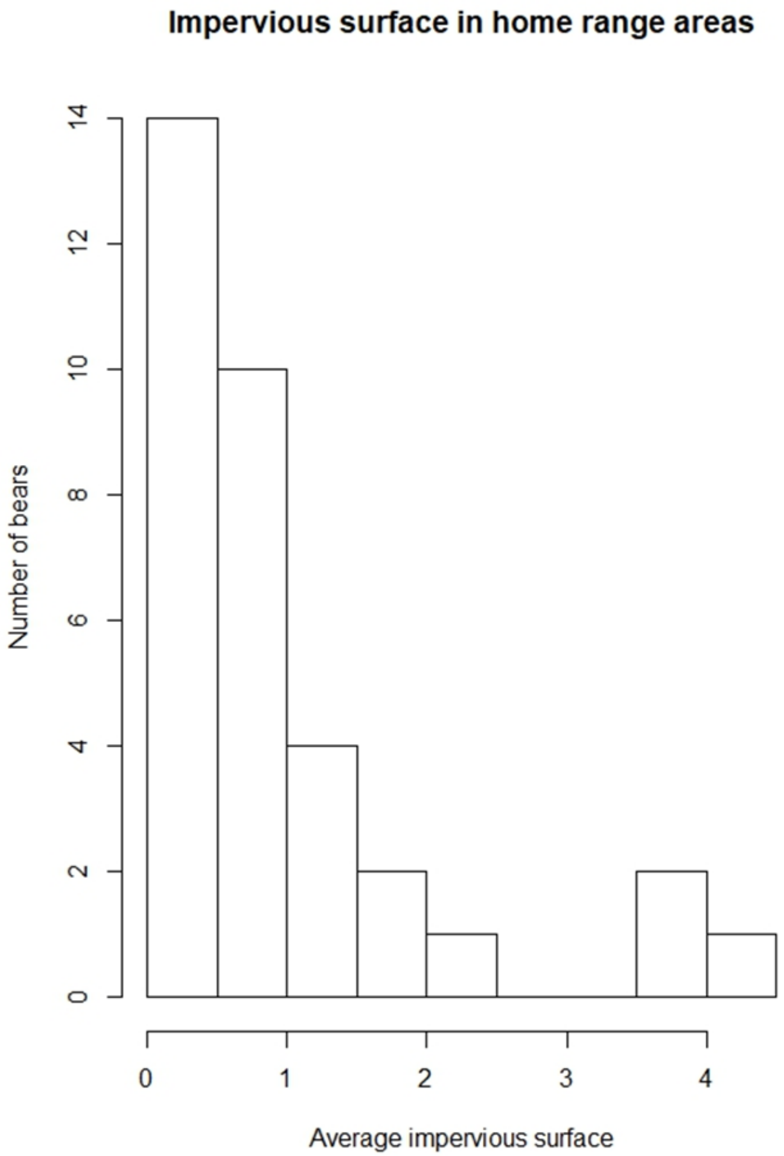

Figure S1. Average percent of impervious surface available within the fall home range areas (1 Sept –30 Nov) for each of the 34 female bears in the *research* group.

Table S1. A summary of each *research* bear's minimum convex polygon (MCP), mean impervious surface (% Imp. surf.) across the fall home range area, and the subgroup where each female was placed based on impervious surface. This subgroup determined the original assumption of food conditioning. The MCP values are reported in kilometers squared (km^2) rounded to the nearest hundredth.

Bear ID	95% MCP	% Imp. surf.	Subgroup
**F600**	45.18	0.59	Wild
**F601**	10.03	0.63	Wild
**F602**	43.17	1.67	Developed
**F603**	136.79	0.26	Wild
**F604**	391.23	0.15	Wild
**F605**	167.96	0.21	Wild
**F606**	96.67	0.84	Wild
**F607**	56.80	0.18	Wild
**F608**	72.83	0.16	Wild
**F609**	48.26	0.16	Wild
**F610**	28.00	0.93	Wild
**F611**	7.50	0.45	Wild
**F612**	32.75	1.24	Developed
**F613**	43.05	0.15	Wild
**F614**	94.28	0.39	Wild
**F615**	11.44	1.06	Developed
**F618**	8.05	3.67	Developed
**F619**	24.92	1.27	Developed
**F620**	33.92	2.30	Developed
**F621**	88.56	1.64	Developed
**F623**	13.18	0.36	Wild
**F624**	71.22	0.40	Wild
**F625**	9.57	0.39	Wild
**F626**	46.42	0.72	Wild
**F627**	5.16	0.74	Wild
**F628**	139.22	0.68	Wild
**F629**	199.94	1.09	Developed
**F631**	8.41	3.90	Developed
**F632**	10.62	0.76	Wild
**F633**	48.74	0.35	Wild
**F634**	12.89	0.41	Wild
**F635**	69.82	0.58	Wild
**F636**	25.05	0.86	Wild
**F637**	8.12	4.46	Developed

Table S2. Individual, mean and SDs of stable isotope ($\delta^{13}C$ and $\delta^{15}N$) values of *anthropogenic* (A) *and management* (C) bears sampled throughout Florida during 2015-2017 and *wild* (B) and *developed* (D) bears sampled in and around Tate's Hell State Forest, Florida, USA, 2016-2017. Subadult age class includes bears aged 1-2.9 years. Diet-year denotes the capture year or prior year for *anthropogenic* and *management* bears, depending on month of capture, and the year prior to capture for *wild* and *developed* bears. LDA classification denotes the two categories (not food conditioned [NFC] or food conditioned [FC]) based on the linear discriminant analysis with leave-one-out cross validation. P-FC is the probability of each individual being food conditioned. While all *anthropogenic* bears were initially assumed to be FC, and all *wild* bears were initially assumed to be NFC, some bears from each subgroup, in bold, were reclassified based on the LDA outputs.

Bear ID	Sex	Age Class	Diet-year	$\delta^{15}N$ (⁰/00)	$\delta^{13}C$ (⁰/00)	LDA Class.	P-FC
**A. Anthropogenic bears captured 2015-2017 (*n* = 28)**							
16766	F	Adult	2014	7.01	−17.86	FC	0.992
16783	M	Adult	2014	6.3	−19.35	FC	0.954
17162	M	Adult	2015	3.75	−24.4	**NFC**	0.03
17190	M	Subadult	2015	6.01	−19.2	FC	0.952
17233	F	Adult	2014	4.01	−23.02	**NFC**	0.148
17355	F	Adult	2015	4.36	−22.01	**NFC**	0.372
17356	M	Cub	2015	5.09	−21.62	FC	0.587
17357	F	Cub	2015	5.83	−22.06	FC	0.606
17485	M	Adult	2015	6.66	−16.75	FC	0.997
17522	M	Subadult	2015	8.66	−22.33	FC	0.829
17523	F	Adult	2015	6.83	−18.98	FC	0.976
17615	M	Subadult	2015	5.41	−20.22	FC	0.85
17617	M	Subadult	2015	7.61	−19.99	FC	0.965
17635	F	Subadult	2016	6.81	−19.16	FC	0.972
17985	M	Adult	2016	6.74	−17.11	FC	0.995
17987	M	Subadult	2015	5.56	−21.23	FC	0.73
18008	F	Adult	2016	7.28	−17.6	FC	0.993
18117	F	Subadult	2016	3.5	−21.42	**NFC**	0.31
18123	F	Adult	2016	7.23	−23.95	**NFC**	0.274
18201	F	Subadult	2016	3.02	−23.04	**NFC**	0.057
18238	M	Adult	2016	6.68	−19.55	FC	0.957
18310	F	Subadult	2016	4.73	−21.35	FC	0.581
18335	F	Adult	2016	6.04	−19.31	FC	0.948
18344	F	Subadult	2016	8.04	−18	FC	0.996
18411	F	Subadult	2015	5.95	−20.83	FC	0.829
18529	M	Adult	2016	6.3	−19.86	FC	0.931
18705	F	Adult	2017	6.07	−19.18	FC	0.954

18744	F	Adult	2017	6.55	−19.96	FC	0.936
Mean				6	−20.33		
SD				1.38	2.02		
**B. Wild bears 2016-2017 (*n* = 24)**							
F600	F	Adult	2015	5.49	−24.26	NFC	0.185
F601	F	Adult	2015	5.06	−23.49	NFC	0.238
F603	F	Adult	2015	4.92	−24.24	NFC	0.134
F604	F	Adult	2015	4.76	−23.4	NFC	0.216
F605	F	Adult	2015	3.68	−20.58	**FC**	0.687
F606	F	Adult	2015	4.79	−23.81	NFC	0.167
F607	F	Adult	2015	4.04	−23.98	NFC	0.098
F608	F	Adult	2015	5.84	−20.4	**FC**	0.899
F609	F	Adult	2015	4.39	−24.52	NFC	0.079
F610	F	Adult	2015	4.14	−21.82	NFC	0.431
F611	F	Adult	2015	3.99	−21.95	NFC	0.382
F613	F	Adult	2015	5.3	−23.84	NFC	0.217
F614	F	Adult	2015	4.8	−24.11	NFC	0.137
F623	F	Adult	2016	3.49	−25.07	NFC	0.029
F624	F	Adult	2016	3.3	−25.14	NFC	0.025
F625	F	Adult	2016	3.68	−24.6	NFC	0.049
F626	F	Adult	2016	3.48	−22.28	NFC	0.258
F627	F	Juvenile	2016	3.48	−25.47	NFC	0.021
F628	F	Juvenile	2016	3.81	−24.72	NFC	0.048
F632	F	Adult	2016	3	−25.1	NFC	0.021
F633	F	Adult	2016	6.91	−24.41	NFC	0.413
F634	F	Adult	2016	5.02	−24.67	NFC	0.104
F635	F	Adult	2016	4.16	−21.77	NFC	0.445
F636	F	Juvenile	2016	3.09	−22.06	NFC	0.261
Mean				4.36	−23.57		
SD				0.96	1.46		
**C. Management bears 2015-2017 (*n* = 17)**							
17160	F	Subadult	2015	4.46	−23.61	NFC	0.156
17166	F	Adult	2015	5.38	−23.87	NFC	0.209
17196	M	Adult	2015	4.77	−23.77	NFC	0.164
17213	F	Subadult	2015	5.73	−19.83	FC	0.911
17240	M	Subadult	2015	6.06	−17.31	FC	0.991
17246	F	Adult	2015	4.3	−23.52	NFC	0.153
17737	M	Adult	2016	6.92	−18.88	FC	0.98
18061	M	Subadult	2016	6.92	−19.70	FC	0.96
18062	M	Adult	2016	6.71	−19.83	FC	0.95
18070	M	Subadult	2016	4.05	−21.74	NFC	0.411

18252	M	Subadult	2016	2.98	−21.93	NFC	0.232
18258	F	Adult	2016	5.47	−19.77	FC	0.901
18265	M	Subadult	2016	4.2	−21.25	FC	0.538
18301	M	Adult	2016	4.83	−20.01	FC	0.833
18322	M	Adult	2016	2.84	−21.16	NFC	0.348
18533	M	Subadult	2016	3.83	−22.69	NFC	0.213
18589	M	Subadult	2017	5.38	−22.30	FC	0.501
Mean				4.99	−21.25		
SD				1.25	1.92		
**D. Developed bears 2016-2017 (*n* = 10)**							
602	F	Adult	2015	5.07	−23.86	NFC	0.18
612	F	Adult	2015	5.79	−22.54	FC	0.515
615	F	Adult	2015	4.22	−22.41	NFC	0.306
618	F	Adult	2016	5.21	−22.56	NFC	0.42
619	F	Adult	2016	5.51	−24.54	NFC	0.14
620	F	Adult	2016	3.51	−24.17	NFC	0.059
621	F	Adult	2016	5	−23.96	NFC	0.162
629	F	Adult	2016	4.24	−23.12	NFC	0.196
631	F	Juvenile	2016	3.55	−23.00	NFC	0.149
637	F	Adult	2016	6.17	−18.20	FC	0.982
Mean				4.83	−22.84		
SD				0.91	1.79		
